# Reconstruction of Bony Defects after Tumor Resection with 3D-Printed Anatomically Conforming Pelvic Prostheses through a Novel Treatment Strategy

**DOI:** 10.1155/2020/8513070

**Published:** 2020-12-01

**Authors:** Wei Peng, Runlong Zheng, Hongmei Wang, Xunwu Huang

**Affiliations:** ^1^Department of Orthopedic Surgery, Eighth Medical Center of Chinese PLA General Hospital, Beijing 100091, China; ^2^Department of Oncology, Eighth Medical Center of Chinese PLA General Hospital, Beijing 100091, China

## Abstract

There has been an increasing interest and enormous applications in three-dimensional (3D) printing technology and its prosthesis, driving many orthopaedic surgeons to solve the difficult problem of bony defects and explore new ways in surgery approach. However, the most urgent problem is without an effective prosthesis and standard treatment strategy. In order to resolve these problems, this study was performed to explore the use of a 3D-printed anatomically conforming pelvic prosthesis for bony defect reconstruction following tumor resection and to describe a detailed treatment flowchart and the selection of a surgical approach. Six patients aged 48-69 years who had undergone pelvic tumor resection underwent reconstruction using 3D-printed anatomically conforming pelvic prostheses according to individualized bony defects between March 2016 and June 2018. According to the Enneking and Dunham classification, two patients with region I+II tumor involvement underwent reconstruction using the pubic tubercle-anterior superior iliac spine approach and the lateral auxiliary approach and one patient with region II+III and three patients with region I+II+III tumor involvement underwent reconstruction using the pubic tubercle-posterior superior iliac spine approach. The diagnoses were chondrosarcoma and massive osteolysis. After a mean follow-up duration of 30.33 ± 9.89 months (range, 18-42), all patients were alive, without evidence of local recurrence or distant metastases. The average blood loss and blood transfusion volumes during surgery were 2500.00 ± 1461.51 ml (range, 1200-5000) and 2220.00 ± 1277.62 *ml* (range, 800-4080), respectively. During follow-up, the mean visual analogue scale (VAS) score decreased, and the mean Harris hip score increased. There were no signs of hip dislocation, prosthetic loosening, delayed wound healing, or periprosthetic infection. This preliminary study suggests the clinical effectiveness of 3D-printed anatomically conforming pelvic prostheses to reconstruct bony defects and provide anatomical support for pelvic organs. A new surgical approach that can be used to expose and facilitate the installation of 3D-printed prostheses and a new treatment strategy are presented. Further studies with a longer follow-up duration and larger sample size are needed to confirm these encouraging results.

## 1. Introduction

The pelvis is a very important component of the skeletal system that helps stabilize and protect the organs in the pelvis. As the three-dimensional (3D) geometry of the pelvic bone is complex, reconstruction of pelvic bony defects following the resection of large pelvic bone tumors poses a great challenge to orthopaedic surgeons worldwide. Historically, surgeons have used massive allografts or autografts in their attempts to reconstruct these bony defects [1, 2]. However, the many shortcomings associated with allografts or autografts, such as malalignment, malrotation, nonunion, rejection, infection, and refracture, significantly limit the application of this technique [3–6].

At present, the functional and anatomical restoration of the pelvis is the most concerning problem associated with reconstruction. With developments in implantation techniques, prosthetic reconstruction after tumor resection has gradually been adopted by many scholars [7–11]. Several kinds of prosthesis can be used for reconstruction, such as a saddle endoprosthesis, [10, 12] an ice cream cone endoprosthesis [8, 13], and a modular hemipelvic endoprosthesis. Saddle and ice cream cone prostheses require a sufficient amount of preserved iliac bone after tumor resection to support the prosthesis, and because they do not provide anatomical reconstruction, they cannot reconstruct the intact pelvic ring or transfer the biomechanical load of the lower limb, leading to a high occurrence of prosthetic instability or looseness. Meanwhile, due to its emphasis on functional recovery, the modular hemipelvic endoprosthesis cannot protect pelvic organs.

With the development of 3D printing techniques that can applied to the clinic, 3D-printed prostheses have been used in the reconstruction of bony defects following complex pelvic bone tumor resection [14]. Moreover, 3D-printed pelvic prostheses have been used to accurately repair bony defects and improve pelvic function [15, 16]. However, the current approach to using 3D-printed prostheses for pelvic reconstruction is still lacking; because there is no standard treatment strategy, an entirely anatomically conforming pelvic prosthesis has not been reported, and there is no suitable approach by which the pelvis can be easily exposed.

Here, we report a novel treatment strategy and an anatomically conforming shape pelvic prosthesis for the treatment of bony defects after pelvic tumor resection. Compared with previously reported prostheses [9, 10, 13], the prosthesis described herein has an improved anatomically conforming shape and differs in its functional design. Our study also focuses more on the strategies of reconstruction than previous studies. To our knowledge, this is the first report of an entirely anatomically conforming hemipelvic prosthesis, a standard treatment strategy for pelvic reconstruction.

## 2. Materials and Methods

### 2.1. Clinical Data

**Figure 1:**
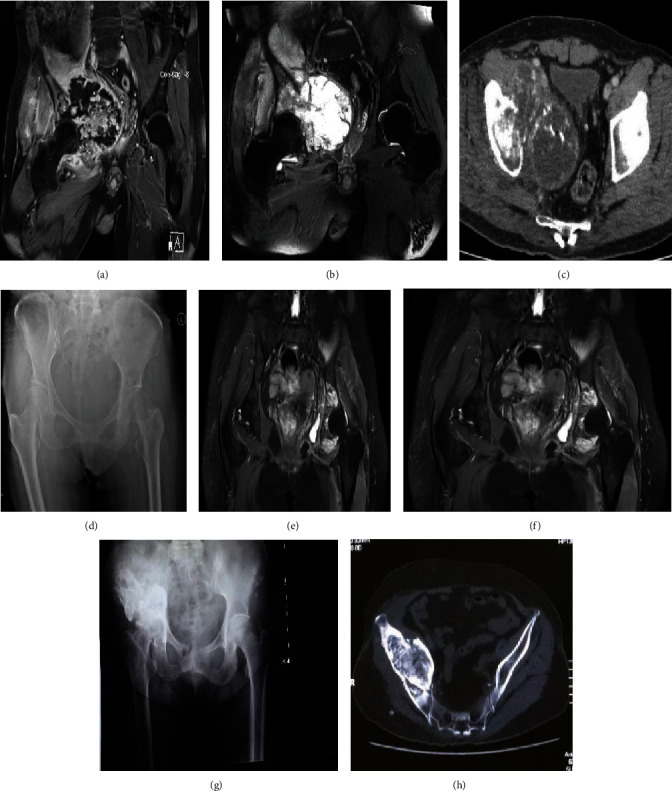
Preoperative radiography, MRI, and CT images. (a) Coronal T2-weighted perfusion MRI scan demonstrating tumor involvement in pelvic regions I+II+III. (b) Coronal T2-weighted MRI scan demonstrating tumor involvement in pelvic regions I+II+III. (c) CT image demonstrating tumor involvement in pelvic regions I+II+III. (d) X-ray image demonstrating tumor involvement in pelvic regions II+III. (e) Coronal T2-weighted perfusion MRI scan demonstrating tumor involvement in pelvic regions II+III. (f) Coronal T2-weighted MRI scan demonstrating tumor involvement in pelvic regions II+III. (g) X-ray image demonstrating tumor involvement in pelvic regions I+II. (h) CT image demonstrating tumor involvement in pelvic regions I+II.

**Figure 2:**
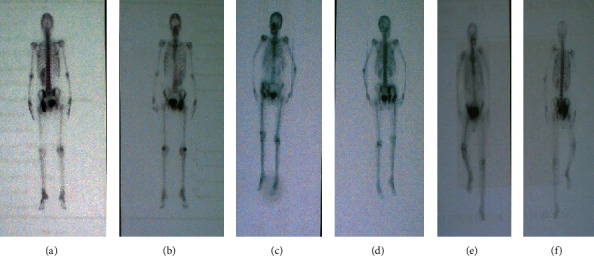
Preoperative electrical capacitance tomography (ECT) bone scans. (a, b) ECT image demonstrating tumor involvement in pelvic regions II+III without distant metastases. (c, d) ECT image demonstrating tumor involvement in pelvic regions I+II without distant metastases. (e, f) ECT image demonstrating tumor involvement.

Between March 2016 and June 2018, six patients with pelvic tumors underwent tumor resection and hemipelvic replacement with a 3D-printed anatomically conforming pelvic prosthesis in Eighth Medical Center of Chinese PLA General Hospital. Inclusion criteria are as follows: (1) pelvic primary low-grade malignant tumors with a good preoperative response to chemotherapy or radiotherapy, with no damage to the external iliac vessels, femoral nerve, sciatic nerve, or main muscles such as the gluteus maximus, and with no tumor metastasis; (2) large osteolytic benign tumors; and (3) pelvic chondrosarcoma, especially around the acetabulum. Exclusion criteria are as follows: (1) metastases from other sites, such as the lung; (2) intolerance to anaesthesia; and (3) malnutrition, a preoperative haemoglobin level below 100 g/l or a plasma albumin level below 30 g/l. There were four male and two female patients with a mean age of 56.00 ± 8.05 years (range, 48-69). All patients underwent preoperative plain radiography of the pelvis, 3D computed tomography (3D-CT) of the pelvis, magnetic resonance imaging (MRI) of the pelvis, and an electrical capacitance tomography (ECT) bone scan (Figures 1 and 2). Needle or incisional biopsy was performed before the operation.

We performed this study after obtaining approval from the Institutional Human Ethical Committee of the 8th Medical Centre, Chinese PLA General Hospital (no 309202008061535).

### 2.2. Workflow Chart of the Surgical Plan

**Figure 3:**
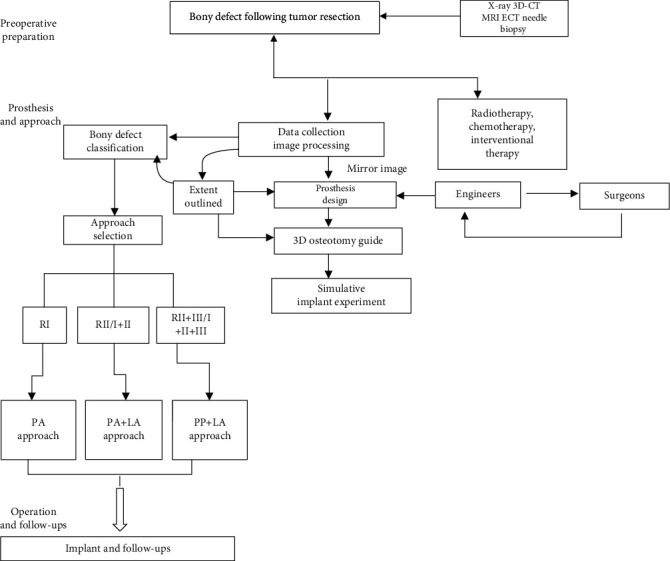
Workflow of a novel treatment strategy for treating pelvic bony defects with 3D-printed prostheses.

The study design consisted of the following preoperative preparation, prosthesis preparation, surgical approach, and follow-up steps (Figure 3), which are described in detail in subsequent subsections. [1] Preoperative preparation included plain radiography of the pelvis, 3D-CT, MRI, an ECT bone scan, and needle biopsy. [2] Preoperative embolization reduces intraoperative bleeding, making tumor resection, osteosynthesis, and implantation of hip prostheses possible. [3] The 3D-printed prostheses were designed and manufactured (through CT data collection and image processing, design of the prosthesis, 3D printing of an osteotomy guide, and an implant simulation experiment). [4] Surgery was performed through the pubic tubercle-posterior superior iliac spine or the pubic tubercle-anterior superior iliac spine approach and/or the lateral auxiliary approach (selected based on the classification of bony defects), comprising osteotomy and implantation. [5] Postoperative management and follow-up were performed.

### 2.3. Data Collection and Image Processing

A set of whole pelvic CT scans comprising the sacrum, ilium, pubis, and ischium was acquired with a 320-detector row CT scanner (Philips Corporation. Japan). Axial images with a slice thickness of 0.5 mm were obtained. The digital images were saved in digital imaging and communications in medicine format and then uploaded into a medical image control system (Mimics 17.0 Software, Materialise Corporation, Belgium) for 3D reconstruction and editing.

### 2.4. Design and Manufacture of the 3D-Printed Anatomically Conforming Pelvic Prosthesis

From our experience in the current study, we have developed the following considerations. [1] The shape of an anatomically conforming prosthesis should aptly fill the bony defect, provide the biomechanical load-bearing capacity for the body, and protect the organs in the pelvis. [2] The prosthetic surface should be firmly integrated with the bone, muscle, and tendon structure of the host, which is a key factor for long-term success [17]. (3)The weight and elastic modulus of the prosthesis need to be taken into consideration so that the prosthesis is not bulky and to avoid stress resulting in a shielding fracture. [4] Guidance of the fixation screws should be effective and accurate.

To produce each of the 3D-printed anatomically conforming pelvis prostheses that addressed a bony defect specific to an individual, the mirror image of the hemipelvis on the unaffected side was combined with the bony defect (as derived from the CT images of each patient), and the prosthesis was then manufactured by 3D printing (Chun Li Zheng Da Medical Instruments Corporation, Ltd., Beijing, China, and Ai Kang Yi Cheng Medical Instruments Corporation, Ltd., Beijing, China) using titanium alloy (Ti6Al4V).

Based on the mirror image principle of 3D printing technology, each complete anatomically conforming prosthesis consisted of an ilium, pubis, and ischium, thus protecting the organs in the pelvis and reestablishing biomechanical loads to recover pelvic function. Of course, the above components were considered when designing the prosthesis, as well as the extent of the bone defect after osteotomy. The obturator foramen was designed as an open ring without the inferior pubic ramus, to facilitate installation and avoid damaging the neurovascular bundle that passes through the obturator foramen. In addition, the ischial tuberosity was designed as a polished surface, providing support and comfort. With no inferior ischial branch, the obturator foramen was designed in an open-loop state that was easy to implant and reduced the risk of injury to vital structures. The anteversion and abduction angles of the acetabulum exactly matched the patient's anatomy. There were three fixed holes at the bottom of the acetabulum to facilitate implantation of the total hip joint. To increase the initial postoperative stability of the reconstruction, the fixed system of the prosthesis included locking screw holes and universal pores.

To reduce the weight of the prosthesis and promote osteointegration, the main structure of the prosthesis body was designed with large holes in a honeycomb-like configuration. The pore diameter of the prosthesis interface with bone ranged from 400 to 450 *μ*m and exhibited a coverage rate of 70% and a thickness of 1.5 mm. A solid structure with a certain strength was designed on the mechanical support portion.

**Figure 4:**
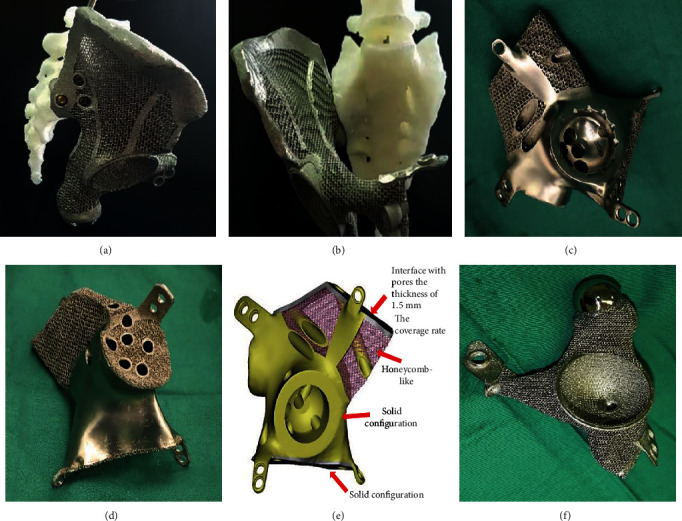
The 3D-printed pelvic prosthesis. (a) Lateral view of a prosthesis designed for the hemipelvis. (b) Anterior view of a prosthesis designed for the hemipelvis. (c) Anterior view of a prosthesis designed for pelvic regions I+II. (d) Posterior view of a prosthesis designed for pelvic regions I+II. (e) Illustration of the prothesis design fabricated by the electron beam melting technique with a porosity of 70% and a pore size of 400 to 450 *μ*m. (f) Anterior view of the prosthesis designed for pelvic regions II+III.

Before surgery, the prosthesis was generated as a whole or part specific for fitting the resected bone or bony defect (Figure 4). Data collection and image processing took three days, while 3D printing prosthesis fabrication, postprocessing cost one week.

### 2.5. 3D-Printed Osteotomy Guide and Implant Simulation Experiment

**Figure 5:**
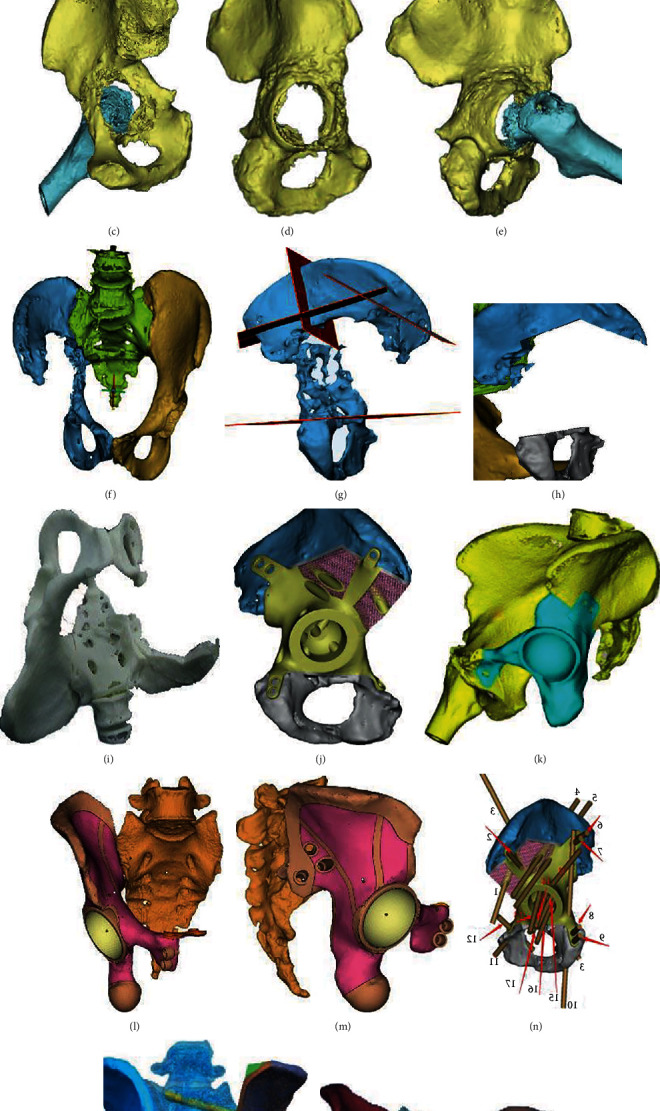
Preoperative planning. (a, b) Embolization was performed to prevent severe hemorrhage. (c–e) 3D-CT image of a tumor in region II. (f–i) The designed osteotomy guide with cutting platforms or slits that matched the planned resection planes. (j–m) Implant trial. (n–p) Drill guide on the implant for drilling the saw paths.

A 3D-printed 1 : 1 plastic model of the pelvic prosthesis, which was produced using the same data and 3D printing technique as the osteotomy guide, was used for preoperative planning and as a reference during the operation. A precise and limited cutting depth of the saw blade was set to prevent the saw from damaging the organs in the pelvis. After a simulation of the osteotomy procedure, a similar simulation experiment was performed for the 3D-printed pelvic prosthesis to determine whether the 3D-printed prosthesis matched perfectly with the residual hemipelvic model (Figure 5).

### 2.6. Surgical Approaches

#### 2.6.1. Preoperative Preparation

Routine enemas were performed twice 12 hours before the operation. After general anaesthesia induction, the patient was placed in a lateral or floating position.

#### 2.6.2. Pubic Tubercle-Posterior Superior Iliac Spine (PP) Approach

**Figure 6:**
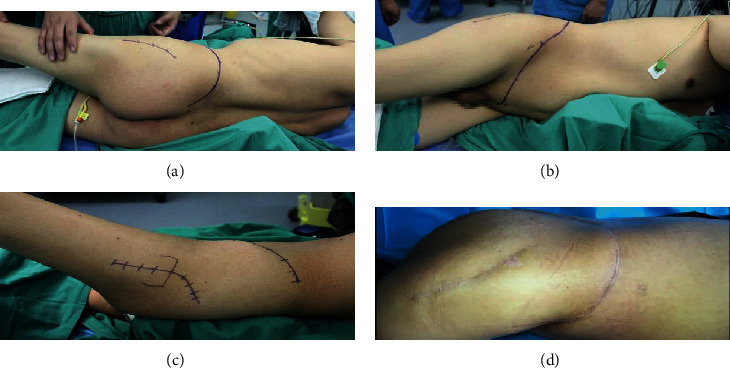
Skin incisions. (a) Posterior view of the pubic tubercle-posterior superior iliac spine approach and the lateral auxiliary approach. (b) Anterior view of the pubic tubercle-posterior superior iliac spine approach and the lateral auxiliary approach. (c) Lateral view of the anterior half of the novel approach and the lateral auxiliary approach. (d) Postoperative wound healing at the one-year follow-up.

For the pubic tubercle-posterior superior iliac spine approach, an incision 60 cm in length was made to expose the tumor focus (Figure 6). Starting from approximately 1 cm above the pubic tubercle, the incision passed through the anterior superior iliac spine and ended at the posterior superior iliac spine. The skin and subcutaneous tissue were incised successively. The gluteus maximus muscle and the external oblique muscle of the abdomen were exposed. As the lateral femoral cutaneous nerve was sometimes encountered at the lateral edge of the incision, care was taken to avoid unnecessary nerve injury. The external oblique muscle was separated from the superficial inguinal canal to the anterior superior iliac spine. The spermatic cord in males or round ligament in females was separated carefully and pulled apart with a rubber band. The anterior sheath of the rectus abdominis muscle was cut to expose the inferior portion of the rectus abdominis muscle. The iliacus muscle was separated from the medial iliac wing. The retropubic space was reached when the rectus abdominis muscle was separated from the pubic symphysis via a transverse incision. After opening the posterior wall of the inguinal canal (which is formed by the internal oblique and transverse abdominal muscles), the inferior epigastric artery was ligated. Then, the fibers of the transverse and internal oblique muscles were separated from the lateral half of the inguinal ligament. The peritoneum was retracted with gauze to expose the femoral artery, femoral nerve, and iliopsoas tendon. After protecting the neurovascular bundle, the medial wall of the acetabulum and the superior pubic ramus were exposed. The gluteus maximus, gluteus medius, and gluteus minimus muscles were peeled from the iliac spine; then, these muscles were carefully retracted inferiorly along with muscles from the anterior compartment of the thigh to expose. The inferior gluteal artery and nerve were avoided to prevent damage. After removing the joint capsule, the femoral neck was sawed from the greater trochanter to a location 1.5 cm above the lesser trochanter. Then, the femoral head was removed.

#### 2.6.3. Lateral Auxiliary (LA) Approach

After exposure of the ilium and pubis as well as part of the ischium, a supplementary incision was performed. For this supplementary posterolateral approach, a longitudinal incision was made with a length of 20 cm from the greater trochanter along the femoral shaft. The tensor fascia lata and gluteus maximus muscles were bluntly separated along the muscle fibers, and then, the sciatic nerve was protected. After removing the attached muscle group from the sciatic tubercle, the whole hemipelvis was freed.

#### 2.6.4. Surgical Approach Selection

The tumors were classified as follows according to the location of the tumor or bony defect in different areas of the pelvis, using the Enneking and Dunham classification [18]: the ilium (region I), acetabulum (region II), and pubis and ischium (region III). Then, based on our experience, the appropriate surgical approach, as described below, was identified and implemented (Table 1). The novel surgical approach, with the goal of resection, was defined by considering the classification of and the extent to which the tumor invaded the pelvis.

1. Region I resection and reconstruction (using the PA approach): for patients with iliac tumors, the operation was performed using the following approach: one incision from the pubic tubercle to the anterior superior iliac spine
2. Region II/I+II resection and reconstruction (using the PA approach plus the lateral auxiliary approach): for patients with acetabular tumors, the operation was performed using the following approach: one incision from the pubic tubercle to the anterior superior iliac spine as well as the lateral auxiliary incision
3. Region II+III/I+II+III resection and reconstruction (using the PP approach + the lateral auxiliary approach)

#### 2.6.5. Osteotomy and Prosthesis Implantation

**Figure 7:**
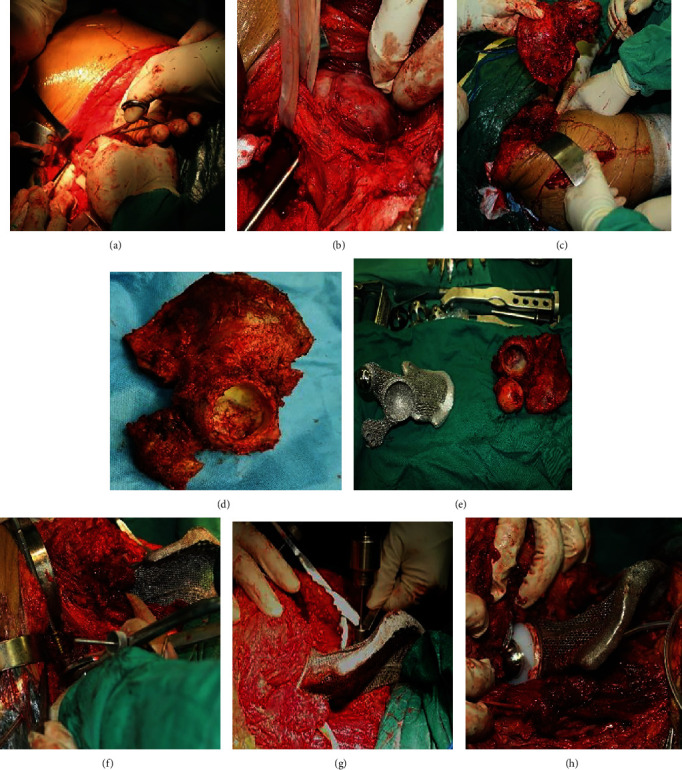
Surgical steps of resection and implantation. (a) An incision was made. (b) The neurovascular bundle was protected. (c) The hemipelvis was removed. (d) The intact hemipelvis. (e) Comparison of the intact hemipelvis and the 3D-printed hemipelvic prosthesis. (f) Implantation of the 3D-printed hemipelvic prosthesis. (g) Fixation of the prosthesis with a drill. (h) Implantation of the total hip prosthesis.

To increase the accuracy of osteotomy and shorten the operative time, a 3D-printed osteotomy guide was developed. The basic principle of the osteotomy guide was designed following the extent of the affected hemipelvis according to the preoperative CT and MRI patient data. The entire process of designing the 3D-printed osteotomy guide was achieved with a preoperatively designed location and 3D-printed cutting guides. The 3D-printing osteotomy guide was fixed at the corresponding region of the bone surface to perform the osteotomy. Under the assistance of the osteotomy guide, the ilium, pubis, and/or ischium was resected. After osteotomy, the 3D-printed anatomically conforming pelvic prosthesis that had been adapted precisely to the bony defect of the pelvis was firmly fixed with cancellous screws and locking screws after drilling via a drill guide. The pubic part of the prosthesis was fixed to the residual part of the pubis or the opposite pubic ramus. The iliac part of the prosthesis was fixed to the iliac crest, the sacroiliac joint, or the residual part of the sacroiliac joint with screws. A polyethylene elevated-rim acetabular liner or a restricted acetabular was cemented to the acetabular rim of the hemipelvic prosthesis for fixation. A cementless femoral stem and metal head were implanted after reaming of the femoral medullary cavity (Figures 7). The surgical field was flushed with a medical pulse irrigator (Beijing Wanjie Medical Device Corporation, Ltd., Beijing, China) to reduce the risk of infection.

### 2.7. Postoperative Management and Main Outcome Measures

During the postoperative period, two silicone drainage tubes were maintained until the drainage volume was less than 50 ml in 24 hours, and second-generation cephalosporin was administered intravenously for seven days. Anticoagulation agents were administered subcutaneously for prophylaxis against deep vein thrombosis for four weeks. Initiation of mobilization was advised when the drainage tubes were removed postoperatively and if there was no pain, no symptoms of instability, and no perioperative complications. All patients underwent postoperative pelvic plain radiography to assess implant orientation and implant failure, such as loosening or fracture, every three months during the first year postoperatively and every six months thereafter. Functional outcome was evaluated by the Harris hip score every month during the first three months of follow-up and every three months thereafter. Preoperative and postoperative pain at rest was assessed using a visual analogue scale (VAS) and the Musculoskeletal Tumor Society (MSTS) score for evaluating patient activity.

### 2.8. Statistical Analysis

Results of VAS score, Harris hip score, follow-up duration, MSTS-93 score, intraoperative blood loss, volume of blood transfused, postoperative drainage volume and operation time were expressed as means ± SD and were analyzed with SPSS Statistics software, version 23.0 (IBM, Armonk, New York, USA). Statistical significance for the results of VAS score and Harris hip score was determined by paired *t*-test. *p* < 0.05 was considered statistically significant.

## 3. Results

### 3.1. Patient Data

Data pertaining to clinical characteristics were recorded—specifically, age, sex, symptoms, duration in months, Enneking and Dunham classification, and diagnosis. Clinical data are summarized in Table 2. The chief complaints were persistent pain in the inguinal area and a fixed local mass in the stomach. X-ray showed osteolytic bone destruction, CT scan showed incomplete bony shell and unclear boundary around the tumor, and soft tissue mass protruded from the bony shell and showed inhomogeneous density. MRI scan showed that the tumor had heterogeneous intensity on T1 and T2 images in five cases, MRI signal intensity of the bone marrow disappeared and had a long T1 and long T2 signal intensity changes in one case, and the radionuclide concentration of tumor foci could be seen on ECT images. According to radiology, Region I and II involvement was found in two patients, region II and III involvement in one patient, and regions I, II, and III involvement in three patients.

### 3.2. Surgical Outcome

**Figure 8:**
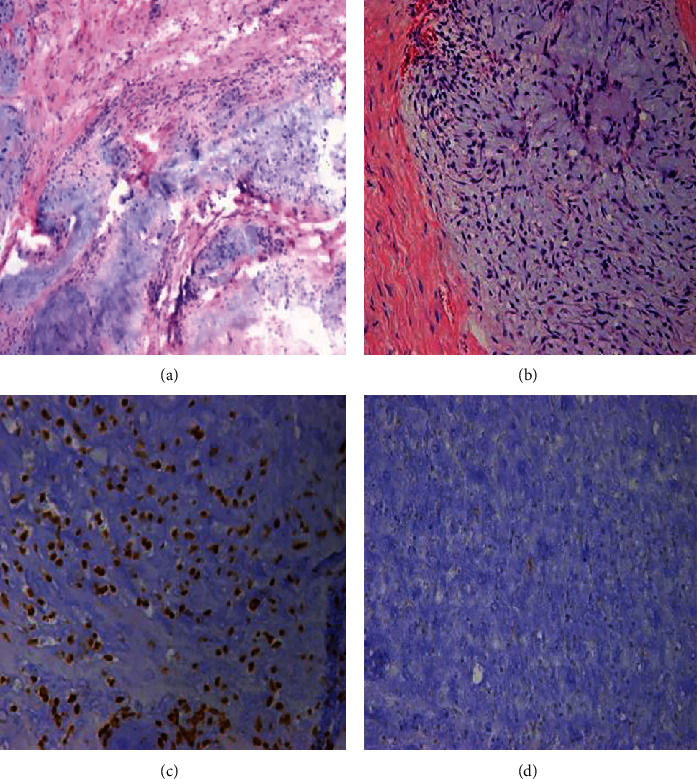
Immunohistochemical pathological evidence from the intraoperative specimen confirms the preoperative diagnosis of chondrosarcoma in a 57-year-old patient. (a) HE ×40. (b) HE ×100. (c) Vimentin ×100. (d) S-100 ×100.

All operations were completed successfully. The five patients with chondrosarcoma, characterized by a large soft tissue mass, underwent wide excision, and the other patient with massive osteolysis underwent marginal excision. Two patients underwent implantation of the 3D-printed prosthesis via the PA approach in addition to the LA approach; the other four patients underwent implantation of the 3D prosthesis via the PP approach in addition to the LA approach. The mean intraoperative blood loss was 2500.00 ± 1461.51 ml (range, 1200-5000), the average volume of blood transfused during surgery was 2220.00 ± 1277.62 ml (range, 800-4080), and the mean postoperative drainage volume was 937.50 ± 474.44 ml (range, 395-1730). The mean operation time was 247.50 ± 103.52 min (range, 135-420 min). Pathological evidence of the intraoperative specimen confirmed the diagnosis from the preoperative biopsy (Figure 8). All cases were studied by pathology correlated with radiology. Among the six patients, five were diagnosed with chondrosarcoma, and the other was diagnosed with massive osteolysis.

### 3.3. Follow-Up Data

**Figure 9:**
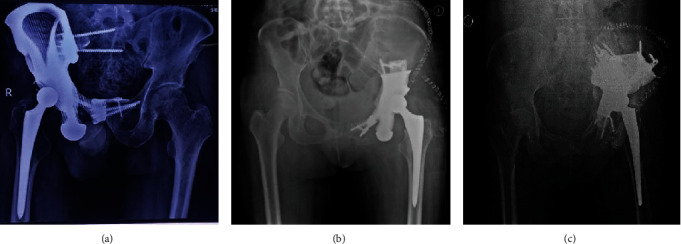
Postoperative X-ray images. (a) Prosthesis for regions I+II+III. (b) Prosthesis for regions II+III. (c) Prosthesis for regions I+II.

After a mean follow-up duration of 30.33 ± 9.89 months (range, 18-42), all patients were alive and without evidence of local recurrence or distant metastases. Hip dislocation, aseptic loosening, bone resorption, periprosthetic fractures (Figure 9), wound infection, and delayed wound healing were not observed in these patients during the short-term follow-up.

**Figure 10:**
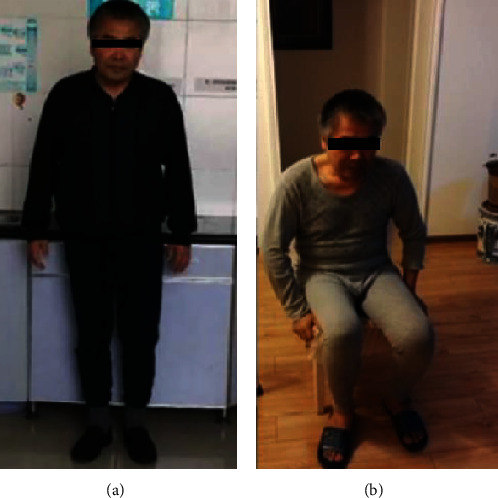
The patient could sit and stand without assistance at the one-year follow-up.

The mean preoperative VAS score was 7.167 ± 1.47 (range, 5-9) one week before operation, and the mean postoperative VAS score was 2.83 ± 1.47 (range, 1-5) at the last follow-up (*t* = 4.914, *p* = 0.004). The mean preoperative Harris hip score was 32.33 ± 11.11 (range, 20-50) one week before operation, and the mean postoperative Harris hip score was 58.17 ± 13.92 (range, 40-78) at the last follow-up (*t* = −7.289, *p* = 0.001). The MSTS score was 19.83 ± 4.26 (range, 15-26) at the last follow-up. The patients were able to walk with crutches after an average of 76.17 ± 10.30 days (range, 62-91) (Table 3, Figure 10).

## 4. Discussion

We establish a novel strategy on reconstruction of bony defects after tumor resection with 3D-printed anatomically conforming pelvic prostheses. With the use of this technology, it will enhance accuracy in preoperative planning and the improvement in outcomes. Herein, we discuss our experience with a 3D-printed prosthesis and address the following: first, how to plan the treatment strategy; second, how to design the prosthesis; and third, how to select the surgical approach.

### 4.1. Planning for the Treatment Strategy

We created a flowchart to illustrate hypothetical management strategies. Based on the results of our study and the flowchart of the study design presented in Figure 3, we propose a similar process for the selection of treatment strategy. With an effective clinical flowchart, it would be beneficial to select surgical indications, simplify the corresponding treatment process, and observe postoperative follow-up. We preliminarily identified multiple possible contributing factors for an effective treatment strategy, including preoperative preparation, tumor classification, and postoperative management. Radiology of data collection played an important role in preoperative preparation.

Good outcomes begin with meticulous planning of the therapy procedure. To ensure surgical accuracy, it is necessary to collect imaging data, such as X-ray, 2D-CT, 3D-CT, MRI, or ECT bone scan data, during the preoperative planning stage. Preoperative lung CT and whole-body isotope bone scans were used to determine the presence of lung and systemic metastases. Invasion of the ureteropelvic segment and important nerve tissues were assessed by preoperative angiography and electromyography. We carried out simulation and guided osteotomy according to the imaging data collected before operation. Application of a 3D-printed osteotomy guide achieves limited exposure and high accuracy and facilitates the desired osteotomy procedure, [19, 20] aiding in precise prosthesis implantation. By using an osteotomy guide, secondary damage to adjacent vessels and nerves can be avoided [21, 22].

It is another concern on how to reduce intraoperative bleeding in preoperative planning. Resection of pelvic tumors causes severe blood loss, even postoperative death [23]. Preoperative tumor vessel embolization can reduce intraoperative bleeding. Blood loss was a major concern in this study, and the estimated average amount of bleeding was approximately 2500 ml, the success of which was attributed not only to the selection of surgical approach and technique but also to the use of preoperative arterial embolization [24]. Selection of the occluding material and the interval between embolization and surgery are keys to the success of embolization [25]. In this study, gel foam was used as an occluding material 1-6 days before operation, which was effective and safe.

In the preoperative planning process, it is very important to select suitable patients with appropriate indications and exclude inappropriate patients with contraindications. A sensitive response to preoperative neoadjuvant chemotherapy and radiotherapy is very important in the treatment of pelvic malignancies, as these types of therapy shrink the tumor volume and clear the tumor boundary [26].

Dislocation is the most common complication in the early postoperative stage. No dislocation or loosening was observed in our patients, which may be associated with the strict postoperative management as shown in our flowchart. This finding could also be attributed to acetabular component positioning [27], soft tissue laxity around the joint [28], and nerve injury. [29] Based on previous experience obtained from the current study, an elevated-rim acetabular liner, a locked hip prosthesis, braces, neutral orthopaedic shoes, and low branch traction were applied in order to help prevent dislocation. The occurrence of hip dislocation was reduced after scar tissue was formed around the hip joint. Previous studies have shown a high risk of infection in patients after undergoing pelvic reconstruction [30]. A medical pulse irrigator and sufficient drainage were applied to reduce the incidence of infection, and no cases of infection occurred in our study during the follow-up period.

### 4.2. Designing the Prosthesis

To obtain satisfactory functional results with reconstruction, restoration of the anatomical integrity of the pelvic ring should be taken into account when designing the prosthesis. Moreover, the selection of the type and extent of pelvic resection informs prosthesis design, including the components, shape, width, and length of the prosthesis. In the present study, the prosthesis was fixed to the remaining ilium (or the sacrum), pubic rami (same side or the other side), and ischial rami by screws through reserved holes, which allowed immediate stability and continuity of the pelvic ring. Besides considering the integrity of the pelvic ring, we design the ischial tubercle of the prosthesis into a polished surface, which can improve the comfort when sitting.

The porous surface structure of the prothesis was designed to reduce the incidence of prosthetic loosening, enhance bone ingrowth, minimize stress shielding, and reduce its weight using electron beam melting technology [17]. Previous studies have reported that this porous structure acts as an osteoconductive scaffold for bone ingrowth and applies a stimulus for bone formation [31]. It has been suggested that porous structures with pore diameters ranging from 400 to 800 *μ*m and high porosity (75%) are the most effective in promoting prosthesis osteointegration [32, 33]. Pobloth et al. reported that a prosthesis designed in a honeycomb-like configuration could minimize stress shielding while ensuring resistance against mechanical failure and increased bone formation [34]. According to these findings, the surface of our 3D-printed prosthesis was designed with a pore diameter of 400 *μ*m and a porosity of 75%. We hypothesized that compared with a smooth surface, this porous structure would enable the well-vascularized soft tissue to better reattach to the prosthesis. In practice, the high postoperative Harris hip score in our study confirmed this hypothesis. Furthermore, we demonstrated the achievement of good radiographic fusion and hip function without any cases of prosthetic loosening or fracture on X-ray at the last follow-up.

Through 3D image processing, safe and dangerous areas of screw fixation can be strictly depicted before surgery to improve safety [35]. A predesigned 3D printing screw hole has been successfully used to drill holes with an accurate orientation and length and to guide screws into the correct position during surgery. The use of preoperative 3D image processing is a safe, effective, and accurate means to avoid the need for repeat detection of the bone defect, increasing the operative time and reducing the incidence of secondary damage. In our study, cement was used to seal the cap of the screws to prevent screw withdrawal. In future research, we plan to use locking screws and holes. Currently, preoperative utilization of the 3D 1 : 1 prosthesis model aid in our understanding of the operation process, shortens the operative time, and reduces the occurrence of complications. Moreover, 3D-printed osteotomy guides have been widely applied in bone tumor resection due to their accuracy [36]. A previous study demonstrated that the maximum error of the 3D-printed osteotomy guide was significantly lower than that of a free-hand osteotomy guide [22]. Under the assistance of a 3D-printed osteotomy guide, intraoperative injury to adjacent blood vessels and nerves can be avoided [21, 22].

### 4.3. Selecting a Surgical Approach

Optimal outcomes are associated with the correct selection of surgical approaches and techniques. Tumor classification is vital, as there is not a single surgical approach that permits safe resection and implantation. The selected approach should also be aimed at avoiding the dissection of important muscle groups, thus reducing the risk of injury to neurovascular bundles. Therefore, the effective surgical approach should be selected according to the tumor classification.

In this novel approach, it is necessary to remove the attachment point of the muscle on the iliac crest and peel away the iliac muscle as well as to avoid serious injury, lateral femoral cutaneous nerve damage, bladder injury, bleeding, extended wound closure time, infection, and other complications. Various surgical approaches (ilioinguinal/modified Stoppa) have been previously reported [37]. Compared with the ilioinguinal approach and the modified Stoppa approach, the novel approach has the following advantages: [1] the muscles are peeled away from the bone as a sleeve together, avoiding damage to external rotation muscles and neurovascular bundles and reducing the risk of infection and [2] due to the satisfactory exposure, it is easy to perform tumor resection and reconstruct the pelvic bony defect with less bleeding.

The volume of blood loss and operative time in this study were partially due to the choice of surgical approach. A long surgical time and a massive blood loss volume might increase the incidence of postoperative infection [9, 21, 33]. Compared with the approach and the modified Stoppa, our novel approach is superior in terms of reducing the operative time and blood loss volume, according to our preliminary evidence. Further studies with larger sample sizes are needed to confirm this hypothesis. In our patients, the average volume of blood loss and the average amount of blood transfused during surgery were lower than many previous reports in which other approaches were used [38–40]. We attribute this reduced bleeding to the selection of the approach. As a qualitative comparison of the operative effectiveness of our approach, the surgical time in our patients was approximately 4 hours, while Gómez-Palomo et al. reported a surgical time of 6 hours using the ilioinguinal approach for internal hemipelvectomy and reconstruction assisted by 3D printing technology in a patient with pelvic sarcoma [41].

### 4.4. Strengths and Weaknesses

To our knowledge, this study is the first to report reconstruction of the hemipelvis using a fully integrated, anatomically conforming hemipelvic prosthesis, or to propose a novel surgical approach for pelvic tumor resection. We also present the treatment strategy. However, this study had some limitations. (1) A comparison group was not included because there were not enough patients in the study, but that the sample size limited the power and also a comparison group was not included. [2] The relatively short follow-up duration did not account for determining late complications in all the patients. [3] There were several drawbacks to the study design. Specifically, large holes in the prosthesis should be utilized according to the anatomical origins of muscles to guarantee accurate fixation and reconstruction of important muscles/tendons, such as the quadriceps femoris and iliac muscles. [4] The stability of the contralateral sacroiliac joint could be affected by the rigid fixation method and osseointegration, a biomechanical performance analysis was not performed in this study, and the prosthesis design should be improved in future studies. [5] The extensive costs associated with 3D printing, the high requirements for the techniques and team members, and the long manufacturing period are also limitations for the extensive application of this approach.

## 5. Conclusions

This is the report of a detailed design for a 3D-printed anatomically conforming hemipelvic prosthesis and a novel treatment strategy in treating the pelvic tumor. In our study, the giant pelvic tumors were resected with large bony defects remaining in need of reconstruction. Reconstruction was achieved with a highly matched, 3D-printed prosthesis with an anatomically conforming design and with surgical techniques that included the novel approach and other supplementary steps to reduce injury. Despite these favorable outcomes, additional studies with long-term follow-up durations are required. Further research will be of value for patients with complex pelvic tumors.

## Figures and Tables

**Table 1:** Definition of the surgical classification and management strategy proposed for each type of resection.

Categorization pelvic involvement			
	R I	R II/I+II	R II+III/I+II+III
Tumor location	Ilium	Acetabulum/ilium and acetabulum	Acetabulum, pubis, and ischium/hemipelvis
Incision			
Start point	Pubic tubercle	Pubic tubercle	Pubic tubercle
End point	Anterior superior iliac spine	Anterior superior iliac spine	Posterior superior iliac spine
Lateral auxiliary incision	No	Yes	Yes
Prosthesis component	Ilium	Acetabulum/ilium and acetabulum	Acetabulum, pubis and ischium/hemipelvis

**Table 2:** Demographics of patients who received a 3D-printed anatomically conforming pelvic prosthesis.

Case	Age	Sex	Classification^※^	Diagnosis	Symptoms	Duration in months
1	48	F	Region I+II	Massive osteolysis	Pain	24
2	57	M	Region I+II+III	Chondrosarcoma	Pain and mass	5
3	69	M	Region II+III	Chondrosarcoma	Pain and mass	6
4	52	M	Region I+II	Chondrosarcoma	Pain and mass	8
5	49	M	Region I+II+III	Chondrosarcoma	Pain and mass	8
6	61	F	Region I+II+III	Chondrosarcoma	Pain and mass	10

^※^According to the Enneking and Dunham classification [18].

**Table 3:** Data related to the operation and follow-up of patients undergoing reconstruction following tumor resection.

Variable	Value
Operation time, mean (min)	247.50 ± 103.52 (range 135-420)
Intra-operative blood loss, mean (ml)	2500.00 ± 1461.51 (range 1200-5000)
Intra-operative blood transfused, mean (ml)	2220.00 ± 1277.62 (range 800-4080)
Post-operative drainage volume, mean (ml)	937.50 ± 474.44 (range 395-1730)
Follow-up duration	30.33 ± 9.89 months (range 18-42)
VAS (pre vs. post)	7.167 ± 1.47 (range 5-9) vs. 2.83 ± 1.47 (range 1-5)
MSTS-93 score, mean	19.83 ± 4.26 (range 15-26)
Harris score (pre vs. post)	32.33 ± 11.11 (range 20-50) vs. 58.17 ± 13.92 (range 40-78)
Average time to walking	76.17 ± 10.30 (range 62-91)

## Data Availability

The dataset supporting the conclusions of this study is available, and we agree to share the dataset.
